# Probing Short‐Range Correlations in the van der Waals Magnet CrSBr by Small‐Angle Neutron Scattering

**DOI:** 10.1002/smsc.202400244

**Published:** 2024-06-13

**Authors:** Andrey Rybakov, Carla Boix‐Constant, Diego Alba Venero, Herre S. J. van der Zant, Samuel Mañas‐Valero, Eugenio Coronado

**Affiliations:** ^1^ Instituto de Ciencia Molecular (ICMol) Universitat de València Catedrático José Beltrán 2 Paterna 46980 Spain; ^2^ Rutherford Appleton Laboratory ISIS Neutron and Muon Facility, Science and Technology Facilities Council Chilton OX11 0QX UK; ^3^ Kavli Institute of Nanoscience Delft University of Technology Lorentzweg 1 2628 CJ Delft The Netherlands

**Keywords:** CrSBr, layered materials, short‐range correlations, small‐angle neutron scattering, van der Waals magnets

## Abstract

The layered metamagnet CrSBr offers a rich interplay between magnetic, optical, and electrical properties that can be extended down to the two‐dimensional (2D) limit. Despite the extensive research regarding the long‐range magnetic order in magnetic van der Waals materials, short‐range correlations have been loosely investigated. By using small‐angle neutron scattering (SANS) the formation of short‐range magnetic regions in CrSBr with correlation lengths that increase upon cooling up to *≈*3 nm at the antiferromagnetic ordering temperature (*T*_N_ *≈ *140 K) is shown. Interestingly, these ferromagnetic correlations start developing below 200 K, i.e., well above *T*_N_. Below *T*_N_, these correlations rapidly decrease and are negligible at low‐temperatures. The experimental results are well‐reproduced by an effective spin Hamiltonian, which pinpoints that the short‐range correlations in CrSBr are intrinsic to the monolayer limit, and discard the appearance of any frustrated phase in CrSBr at low‐temperatures within the experimental window between 2 and 200 nm. Overall, the obtained results are compatible with a spin freezing scenario of the magnetic fluctuations in CrSBr and highlight SANS as a powerful technique for characterizing the rich physical phenomenology beyond the long‐range order paradigm offered by van der Waals magnets.

## Graphical abstract

The correlation length of the layered magnetic semiconductor CrSBr is measured by small‐angle neutron scattering (SANS). The ferromagnetic correlations start developing below 200 K, i.e., well above *T*_N_ (140 K), with a length of *≈*3 nm at *T*_N_. The experimental results are well‐reproduced by an effective spin Hamiltonian, which pinpoints that the short‐range correlations are intrinsic to the monolayer limit.

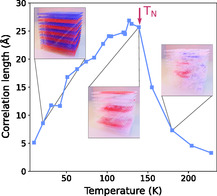

## 1 Introduction

van der Waals (vdW) magnets are a broad family of layered materials that offer a versatile platform for addressing both fundamental questions in low‐dimensional magnetism as well as applied developments in areas such spintronics, magnonics, or data storage since the magnetic layers can be used as building blocks for the fabrication of magnetic vdW heterostructures.^[^ 1, 2, 3 ^]^ Thanks to their rich chemical composition, different magnetic ground states can be found including conventional ferromagnetism (e.g., Fe_3_GeTe_2_ or Cr_2_Ge_2_Te_6_)^[^ 4, 5 ^]^ or antiferromagnetism (e.g., FePS_3_ or NiPS_3_),^[^ ^6^ ^]^ as well as more exotic behaviors such as frustrated magnetism (e.g., CeSiI),^[^ ^7^ ^]^ quantum spin liquids (e.g., RuCl_3_ or 1T‐TaS_2_),^[^ 8, 9, 10 ^]^ or different classes of metamagnetism (e.g., CrI_3_, CrPS_4_ or CrSBr).^[^ 11, 12, 13 ^]^ The structural features of these layered vdW materials—which typically shows strong exchange interactions within the layers but very weak interlayer interactions—provide a unique situation in which the emergence of long‐range magnetic order is strongly dependent on the spin dimensionality.^[^ ^14^ ^]^ In this regard, the role of short‐range correlations coupling electronic and magnetic degrees of freedom is fundamental for understanding the properties of these materials. For instance, short‐range correlations have been related to an enhancement of the thermoelectric properties,^[^ ^15^ ^]^ the appearance of quantum phase transitions^[^ ^16^ ^]^ and even to the origin of high‐temperature superconductivity.^[^ ^17^ ^]^ However, most of the experimental efforts regarding van der Waals magnets have focused on the long‐range magnetic ordered phase, being the quantification of the short‐range correlations relegated to a secondary place, likely due to the lack of proper experimental techniques able to its quantification.^[^ ^14^ ^]^ Interestingly, thanks to the weak inter‐layer interactions in vdW materials, all these properties can be studied down to the 2D limit and even controlled by proximity effects or twisting of the layers.^[^ 18, 19, 20 ^]^

**Figure 1:**
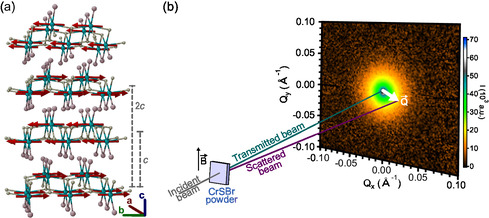
CrSBr crystal structure and small‐angle neutron scattering (SANS) experimental configuration. a) Crystal structure of the layered vdW magnet CrSBr. The chromium, sulphur, and bromine atoms are represented as cyan, yellow, and pink balls, respectively. In the ordered state, the spins within each single layer couple ferromagnetically—spins represented as red arrows—pointing along the *b*‐axis. The ferromagnetic layers couple antiferromagnetically among them along the *c*‐axis. b) SANS experiment schematics in a transmission mode. The intensity of the scattered beam is recorded in a 2D detector (an example at 300 K is shown).

In this work, we consider the layered magnetic semiconductor CrSBr, which has gained recent interest due to the interplay between its magnetic, optical, and electrical properties down to the 2D limit.^[^ 21, 22, 23, 24, 25, 26 ^]^ This material is formed by ferromagnetic layers that couple antiferromagnetically (**Figure** **1**a) undergoing a long‐range magnetic ordering at *T*_N_≈140 K.^[^ ^21^ ^]^ In addition, upon the application of moderate magnetic fields, it is possible to reorient the spin of the layers (at 10 K: 0.6 T, 1 T, and 2 T for fields applied along the *b*, *a*, and *c* axes, respectively).^[^ ^12^ ^]^ Previous muon, heat capacity and magneto‐transport experiments have shown the existence of short‐range correlations in CrSBr already below 200 K,^[^ 12, 23, 27, 28 ^]^ which are highly relevant regarding the light‐matter interaction.^[^ 29, 30, 31, 32 ^]^ However, due to the emergence of long‐range order at lower temperatures, it was not possible to quantify these short‐range correlations. Here, we employ small‐angle neutron scattering (SANS) in order to determine the correlation length of the short‐range fluctuations, as well as its temperature dependence. In the context of magnetism, SANS is a technique able to resolve structures on a length scale between a few and a few hundred nanometers, thus being able to resolve magnetic short‐range correlations, as already demonstrated,^[^ 33, 34, 35, 36 ^]^ while being insensitive to the long‐range ones.^[^ 37, 38, 39, 40 ^]^ Our results show the appearance of short‐range correlations below ≈200 K, characterized by correlations lengths up to *≈*3 nm at *T*_N_, and highlight SANS as a powerful technique for characterizing vdW magnets.

## 2 Results and Discussion

Crystals of CrSBr are grown by a solid‐state reaction (see Experimental Section). CrSBr crystallizes in an orthorhombic space group, characterized by *α* = *β* = *γ* = 90°, *a* = 3.512 Å, *b* = 4.762 Å and *c* = 7.962 Å,^[^ ^12^ ^]^ being *c* related to the distance between the layers (Figure 1a). SANS experiments are performed on a CrSBr powder sample in a transmission geometry (see Experimental Section). As sketched in Figure 1b, the incident neutron beam scatters in the sample and the diffuse magnetic scattering is recorded in a 2D detector. All 2D neutron data are shown in the Supporting Information Section S1. Then, the SANS pattern is obtained by integrating over each azimuthal angle around the center of diffraction, with an experimental detection in the 0.004–0.7 Å^−1^ *Q* range, being *Q* the wavevector. Magnetic fields are applied perpendicular to the incident neutron beam.

**Figure 2:**
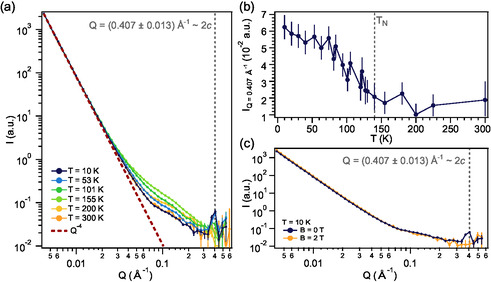
Antiferromagnetic order in CrSBr probed by SANS. a) SANS pattern at selected temperatures (the whole dataset is presented in the Supporting Information Figure S1a). At low temperatures, a Bragg peak is observed at *Q* = 0.407 Å^−1^≈2*c* as a consequence of the antiferromagnetic order. b) Thermal dependence of the SANS intensity at *Q* = 0.407 Å^−1^, where an enhancement of the signal is observed below the Néel temperature (*T*_N_ ≈ 140 K). c) SANS spectra at *T* = 10 K. The peak at *Q* = 0.407 Å^−1^ vanishes at high applied magnetics fields due to the spin‐reorientation of the layers.

SANS patterns at different temperatures are shown in **Figure** **2**a (the complete dataset is shown in Supporting Information Section S1). At low momentum transfer, the pattern follows a *Q* ^−4^ dependence for the whole temperature range steaming from the powder nature of the sample, deviating at *Q* ≈ 0.025 Å^−1^ and flattening above *Q* ≈ 0.3 Å^−1^. Comparing the spectrum at high temperature (*T* = 300 K; orange color in Figure 2a) and low temperature (*T* = 10 K; dark blue color in Figure 2a), the only remarkable difference is the appearance of a Bragg peak at *Q*≈0.407 Å^−1^ at low temperatures. This peak arises due to the antiferromagnetic interlayer coupling (Figure 1a). In fact, taking into account the Bragg relation *Q* = 2π/*d*, *d* can be estimated to be 15.4 ± 0.5 Å, which matches well with a doubling of the cell along the *c*‐axis. The thermal dependence of the intensity of this peak (Figure 2b) corroborates its magnetic nature as it increases below *T*_N_. In addition, application of an external field suppresses this peak since the spins are reoriented along the applied field and, therefore, the antiferromagnetic structure vanishes (Figure 2c).

**Figure 3:**
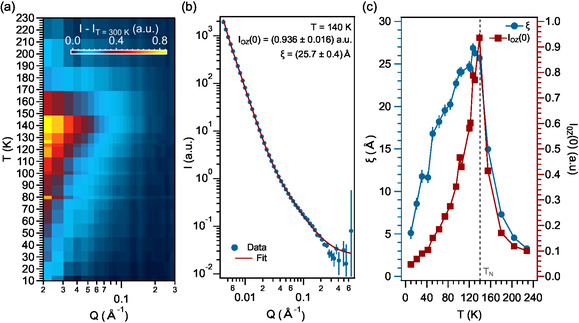
Thermal dependence of the short‐range correlations in CrSBr probed by SANS. a) Thermal dependence of the magnetic contribution of the SANS signal after removing the structural component (*I*_T_ = 300 K). b) SANS signal at *T* = 140 K fitted following an Ornstein–Zernike law, yielding to an estimate of the volume fraction, *I*_OZ_, and correlation length, *ξ* (see text for details). c) Thermal dependence of the volume fraction and correlation length. The Néel temperature is marked as a grey dashed line. The complete set of fittings is presented in the Supporting Information Section S2.

Both spectra at high and low temperatures (Figure 2a) exhibit almost an identical *Q* dependence, except for the appearance of the Bragg peak, as expected for a long‐range ordered antiferromagnet. Interestingly, the major variations in the spectra are observed in the range 0.025–0.3 Å^−1^ at intermediate temperatures (Figure 2a and Supporting Information Figure S2a). For a better visualization of these differences, we show in **Figure** **3**a the magnetic contribution of the SANS signal by subtracting the structural component (in our case, the spectra at 300 K in the high‐temperature paramagnetic phase).^[^ ^41^ ^]^ The complete *Q*‐range is presented in the Supporting Information Figure 2b. While lowering the temperature, the SANS magnetic contribution is already detectable at 200 K, i.e., 60 K above *T*_N_, exhibiting its maximum at *T*_N_ and starting to decrease upon further cooling down (Figure 3a). An example of the SANS spectrum is shown in Figure 3b. We determine the correlation length, *ξ*, based on an Ornstein–Zernike analysis, following a well‐established phenomenological method in SANS as previously reported^[^ 42, 43, 44 ^]^ (see Experimental Section). From the fit (Figure 3b), *ξ* is estimated to be in the order of 3 nm at 140 K.

By performing similar fittings at different temperatures (Supporting Information Section S2), the thermal dependence of *ξ* and the intensity scaling (related to the volume fraction of the correlated regions, assuming that the net magnetic moment is constant) is determined (Figure 3c). *ξ* (blue dots in Figure 3c) increases below 225 K up to a maximum around *T*_N_. Below *T*_N_, *ξ* diminishes and, at temperatures lower than 40 K, the magnetic signal in the studied region decreases very quickly due to the reduction in the volume fraction of the correlated region. This fact is better observed in the intensity scale (red squares in Figure 3c). At high temperatures, *I*_OZ_(0) is almost zero, indicating that the magnetic contribution to the SANS signal is negligible. Below 200 K, *I*_OZ_(0) increases and reaches its maximum at *T*_N_. Below *T*_N_, *I*_OZ_(0) decreases rapidly (by a factor of 3) in the 80–140 K range and, finally, is suppressed below 40 K (thus, a short‐range frustrated magnetic state in CrSBr, as it could be speculated based on the magnetic exchange interactions,^[^ ^23^ ^]^ is not likely to occur at very low‐temperatures). Overall, the observed magnetic behavior is in agreement with a spin‐freezing scenario occurring in CrSBr, as previously suggested by magnetization and muon experiments,^[^ ^23^ ^]^ where only long‐range interactions become relevant at low‐temperatures and, therefore, are not detected in our SANS signal. In contrast with previous observations, where a slowing down of the magnetic fluctuations was observed below *≈*100 K, we observe a rapid suppression of these short‐range correlations just below *T*_N_, becoming negligible below *≈*40 K. Summarizing, we observe correlated regions with a net magnetic moment well above the ordering temperature, that increase while approaching the ordering temperature and are characterized by a correlation length in the order of 3 nm at *T*_N_. By cooling below *T*_N_, these magnetic fluctuations decrease, as observed by the diminishment of the intensity scaling, *I*_OZ_(0).

**Figure 4:**
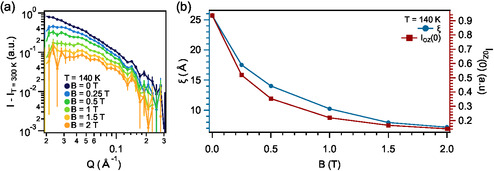
Field dependence of the short‐range correlations in CrSBr at *T* = 140 K. a) Magnetic contribution of the SANS signal at different applied magnetic fields after removing the structural component (*I*_T_ = 300 K). b) Field dependence of the volume fraction and correlation length. The complete set of fittings is presented in the Supporting Information Section S2.

As a secondary fingerprint of the short‐range correlations, we consider the role of applying an external magnetic field. In **Figure** **4**a, we show the magnetic contribution to the SANS signal at *T*_N_ upon the application of different external magnetic fields, observing a suppression of the signal as the field is increased. By performing the same analysis as discussed above, we determine *ξ* and intensity scaling (Figure 4b), which diminish as the applied field is increased. A similar field dependence is observed at 150 K (Supporting Information Section S2). On the contrary, no field dependence in the region between 0.02 and 0.3 Å^−1^ is observed at 10 K (Supporting Information Section S2), in agreement with a scenario with only long‐range order at low temperatures and, therefore, not sensitive by SANS.

For modeling the short‐range correlations in bulk CrSBr, we employ the following effective spin Hamiltonian:

(1) $$H=\sum_{i\neq j}J_{ij}{\vec{S}}_{i}\cdot {\vec{S}}_{j}+\sum_{i\neq j}{\vec{D}}_{ij}\cdot \left({\vec{S}}_{i}\times {\vec{S}}_{j}\right)+\sum_{i}{\vec{S}}_{i}A_{i}{\vec{S}}_{i}+H_{dd}$$

**Figure 5:**
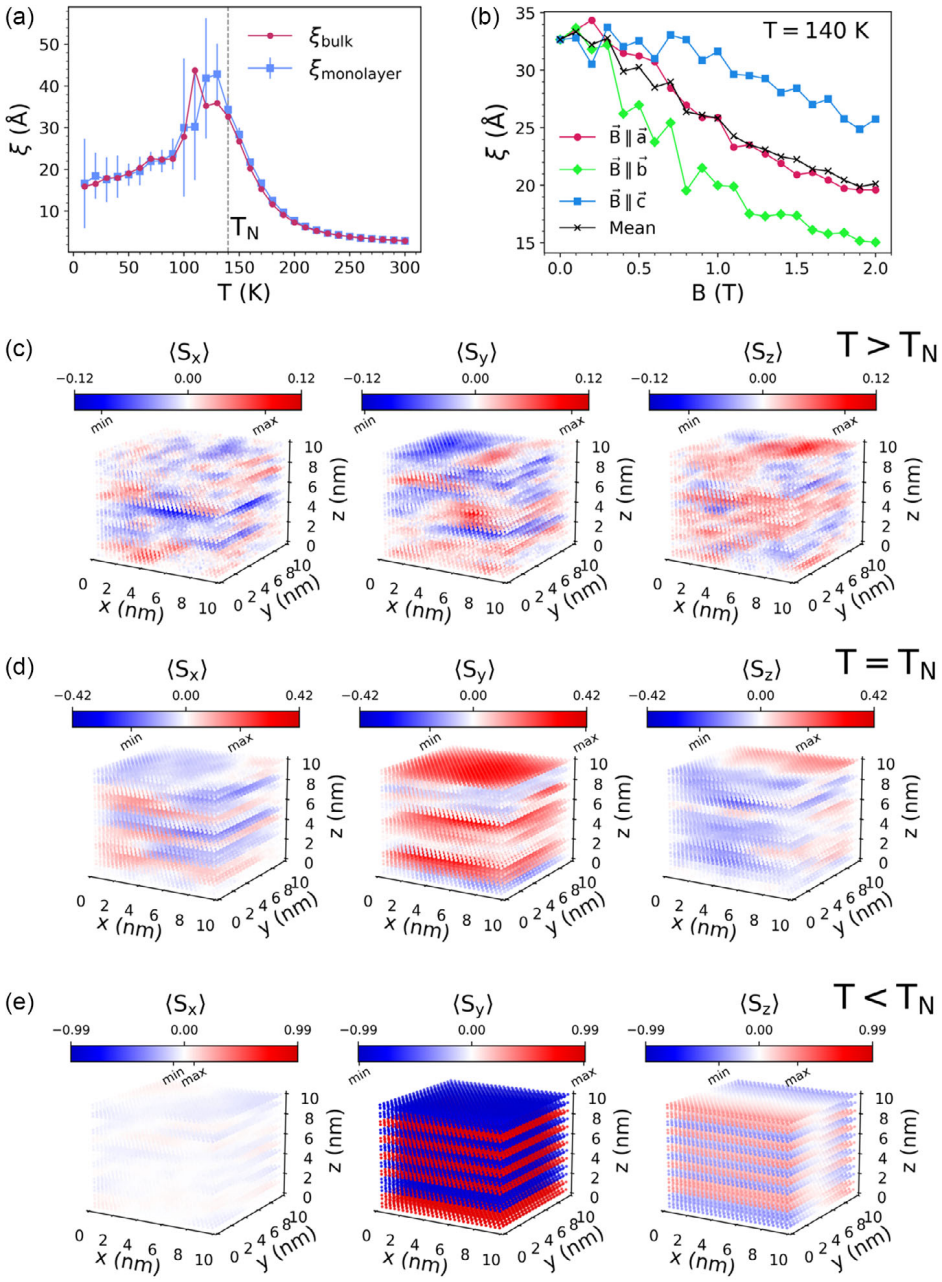
Simulated thermal dependence of the microscopic magnetization in CrSBr. a) Temperature dependence of the correlation length for the intralayer pairs of bulk sample (red circles) and for monolayer sample (blue squares). The error bars for the monolayer indicate one standard deviation. b) Field dependence of the correlation length at 140 K for the intralayer pairs of bulk sample. c–e) Equilibrium spin distribution of the microscopic magnetic moments in the simulated bulk sample in the paramagnetic phase (*T* = 170 K, panel c), at the transition temperature (*T* = 140 K, panel d) and in the ordered phase (*T* = 20 K, panel e). Colors indicate the value of the corresponding magnetization component from minimal (blue) to maximal (red) value, normalization is the same for all components. Minimum and maximum values of each individual component are noted below the color bars. The *a* crystallographic axis of the material is oriented along *x*; *b*—along *y*; *c*—along *z*.

The first term describes the bilinear exchange interactions, where *J*_*ij*_ is an isotropic exchange parameter up to seven neighbor's order in the layers^[^ ^45^ ^]^ (extracted from inelastic neutron scattering experiments) and up to second neighbor's order between the layers (taken from first‐principles calculations).^[^ ^46^ ^]^ The second term takes into account the antisymmetric anisotropic Dzyaloshinskii–Moriya interaction (DMI) that are allowed for some bonds of the CrSBr based on its symmetry (following the first‐principles calculations).^[^ ^46^ ^]^ The third term reflects the triaxial on‐site anisotropy with the values adopted from microwave absorption spectroscopy measurements with A_i_ being a diagonal matrix.^[^ ^47^ ^]^ The last term accounts for magnetic dipole–dipole interactions. For every temperature and magnetic field, we calculate the spin‐spin correlation function from the result of the Monte‐Carlo sampling as implemented in the VAMPIRE computational package.^[^ ^48^ ^]^ The calculated correlation function for bulk exhibits an exponential decay (Supporting Information Section S3) and is separated into the interlayer and intralayer contribution due to the relatively small exchange between the layers of CrSBr (in the range of 10^−3^ meV) as compared to the intralayer ones (in the range of 1 meV), indicating that the 2D intralayer short‐range correlations are the main ones in this material.^[^ ^45^ ^]^ The intralayer term dominates the exponential decay at each temperature while the interlayer contribution remains close to zero for all temperatures and exhibits little or none temperature and field dependence, thus pointing toward the appearance of long‐range interlayer antiferromagnetic interactions only below *T*_N_. By fitting the correlation function (see Experimental Section), we obtain the computed correlation length as a function of temperature (**Figure** **5**a) and magnetic field (Figure 5b), reproducing well our experimental observations. To further illustrate the key role of the intralayer interactions, we compute as well the thermal dependency of the correlation length for a monolayer (blue squares in Figure 5a). Compared to the bulk case (red dots in Figure 5a), the bulk thermal dependency of the correlation length mimics the behavior of the monolayer, reflecting the major role of the intralayer interactions and highlighting that the short‐range correlations in CrSBr arise from the monolayer.

Regarding the magnetic field dependence at *T* = 140 K (Figure 5b), we apply the field along the three main crystallographic axes in three separate simulations. Fields along the medium (*a*) or hard (*c*) axis result in a delayed decay of *ξ* if compared with the field aligned along the easy (*b*) axis (Supporting Information Section S3). Since, experimentally, in a powder sample all directions are present, we consider as well the average trend. Overall, the dynamic correlations are suppressed as the field is increased due to the appearance of long‐range order, in agreement with the experimental result (Figure 4b). Finally, we consider the underlying spin textures appearing in CrSBr while cooling down (Figure 5c–e). First, above *T*_N_, the calculated *ξ* increases as the sample is cooled down, which corresponds to the formation of correlated domains (Figure 5c), while the long‐range order is not present. It reaches the maximum around the phase transition temperature (Figure 5d) and, then, it decreases and saturates at low temperatures, where the long‐range order dominates (Figure 5e). In the region below the phase transition, long range order is established and, therefore, the dynamical correlations are effectively suppressed, as evidenced from the results of the simulations.

## 3 Conclusion

In this work, we have probed the role of short‐range correlations in the van der Waals magnet CrSBr by SANS experiments. Our experimental results quantify the short‐range magnetic fluctuations in CrSBr, which are characterized by a correlation length in the order of 3 nm at the ordering temperature, *T*_N_, and confirm the antiferromagnetic ordering below 140 K as well as the absence of frustrated magnetic states at low‐temperatures. These correlations exhibit an interesting thermal dependence since they are already present below 200 K—that is, well above *T*_N_ —, they exhibit a maximum at *T*_N_ and, then, decrease rapidly while cooling down, being absent at low‐temperatures. In accordance, the application of an external field suppresses these fluctuations. In addition, these experimental observations are well reproduced by a theoretical model based on an effective spin Hamiltonian, highlighting that the appearance of short‐range correlations are intrinsic to the monolayer limit. Overall, our results are in accordance with a spin‐freezing scenario in CrSBr, where the magnetic fluctuations cease while cooling down, and highlight SANS as an optimal technique for characterizing the rich physical phenomenology occurring in vdW magnets beyond the conventional long‐range order picture.

## 4 Experimental Section

4.1

4.1.1

##### Crystal Growth

Crystals of CrSBr are grown by solid‐state techniques, as previously reported by some of us.^[^ ^12^ ^]^ The crystal structure is verified by powder and single‐crystal X‐ray diffraction together with the elemental composition by energy‐dispersive X‐ray spectroscopy (EDS).

##### SANS Measurements

SANS experiments are performed at the Larmor instrument (ISIS neutron and muon source, UK) on 115 mg of CrSBr powder sample. The coherence length of the neutron beam is tens of micrometers, but at least higher than 57 nm, as experimentally probed.^[^ ^43^ ^]^ The incident neutron beam scatters in the sample in a transmission geometry, being the diffuse magnetic scattering recorded in a 2D detector (sample‐detector distance is 4 m, with wavelengths from 0.9 to 13 Å). The signal is integrated over each azimuthal angle around the center of diffraction, with an experimental detection in the 0.004–0.7 Å^−1^ *Q* range, being *Q* the wavevector. SANS spectra are recorded as a function of temperature (10–300 K) and magnetic field (0–2 T), being the magnetic field applied perpendicular to the incident neutron beam. No significant thermal dependence of the SANS pattern is expected unless some structural or magnetic inhomogeneities in the range between 2 and 200 nm—that is, within our experimental sensitivity—start developing.^[^ ^42^ ^]^ The neutron data reduction (including conversion of the Time of Flight and corrections for accounting the background scattering and transmission) is performed using the Mantid software.^[^ ^49^ ^]^ Data fits are done with SasView application (http://www.sasview.org/, using dQ Data instrumental smearing). The data is fitted considering two different approaches. In the first approach (Supporting Information Section S2a), we fit the spectra at 300 K (high‐temperature paramagnetic phase) to a power law, being $I_{T=300K}\left(Q\right)=\frac{I_{P,\;300K}}{Q^{4-n,300K}}+B_{300K}$, where *I*_P_ is a Porod scale term (a particularly common dependence which main contributions are the divergence of the incident neutron beam as well as the Porod scattering from interfaces such as powder grain surfaces and grain boundaries, applicable to our powder measurement),^[^ ^36^ ^]^ *n* is an exponent and *B* is a Q‐independent background constant. Then, we employ the relationship $I_{T\neq 300K}\left(Q\right)=\frac{I_{\text{OZ}}\left(0\right)}{1+{\left(\xi Q\right)}^{2}}+\frac{I_{P,\;300K}}{Q^{4-n,300K}}+B$, where *I*_OZ_(0) is the Ornstein–Zernike intensity scaling and *ξ* is the correlation length. In the second approach (Supporting Information Section S2b), we consider $I\left(Q\right)-I_{300K}\left(Q\right)=\frac{I_{\text{OZ}}\left(0\right)}{1+{\left(\text{ξQ}\right)}^{2}}+\frac{I_{P}}{Q^{4}}+B$. We note that both approaches are compatible between them, although the second one yields to a correlation length with larger error bars at low temperatures, which arise from an overparametrized fitting due to the absence of correlations within our experimental window range resolution. Despite being *ξ* constant below *T*_N_, the absence of correlations is accounted by the suppression of *I*_OZ_(0). Fits shown in the main text are obtained with the first approach. All fitted data following both approaches are shown in the Supporting Information Section S2.

##### Computational Details

Spin‐spin correlation function is calculated from the result of the Monte‐Carlo sampling as implemented in the VAMPIRE computational package.^[^ ^48^ ^]^ The computation is performed considering a 10 × 10 × 10 (nm) sample with open boundary conditions. The lateral size of the simulated sample is chosen in a balance of covering the measured range of the correlation length and the computational cost of the simulation. 10^5^ Monte‐Carlo steps are computed for each temperature and field with first 3·10^4^ reserved for the thermalization. From the microscopic spin distribution, the spin‐spin correlation function $g\left(r_{i},r_{j}\right)=\langle {\vec{S}}_{i}{\vec{S}}_{j}\rangle -\langle {\vec{S}}_{i}\rangle \langle {\vec{S}}_{j}\rangle$ is computed for each temperature, where ⟨…⟩ denotes the canonical ensemble average. Next, the distance‐dependent function $g\left(r_{i}-r_{j}\right)$ is computed by averaging over the pairs with the same distances. Finally, the correlation length is computed assuming the exponential decay of the spin‐spin correlation function over distance. In particular, we consider the exponential fit A·*e* ^−*r*/*ξ*^ for the intralayer part of the correlation function, where A is the amplitude, r the distance, and ξ the correlation length. In order to account for the surface effects only the pairs with the distance smaller than 5 nm are considered for the fit. For constructing the spin Hamiltonian, the intralayer isotropic exchange parameters are extracted from inelastic neutron scattering experiments.^[^ ^45^ ^]^ The interlayer isotropic and intralayer DMI parameters are taken from first‐principles calculations.^[^ ^46^ ^]^ The on‐site anisotropy values are adopted from the results of the microwave absorption spectroscopy measurements.^[^ ^47^ ^]^ We remark that the notation of the spin Hamiltonians differs between sources; thus, the values are converted during the construction of the Hamiltonian (see Supporting Information Table S1 for details). After the construction of the Hamiltonian all parameters are scaled by a factor 1.5 in order to match the experimental value of *T*_N_. In the calculations, the magnetic alignment of the layers does not follow the sequence expected for a pure A‐type antiferromagnet. Still, the total balance of the spin‐up and spin‐down layers is preserved (thus out of 13 layers calculated in Figure 5c 6 are spin‐up and 7 are spin‐down). This result is not unexpected, as the interlayer exchange parameters are *≈*1000 times smaller than the intralayer ones. The intralayer correlation length saturates at the value *≈*2 nm below the phase transition, which is comparable with the result of the measurements. However, it is less stable in the vicinity of the phase transition. This suggests that the description of the equilibrium properties near the phase transition with the Monte‐Carlo simulations may require considerably more iteration steps, which is not feasible from the computational point of view for the bulk.^[^ ^50^ ^]^ For the monolayer case, we start the Monte‐Carlo sampling from 20 different random initial spin configurations and estimate the mean value and standard deviation of the correlation length for each temperature.

## Conflict of Interest

The authors declare no conflict of interest.

## Supporting information

Supplementary Material

## Data Availability

The data that support the findings of this study are available from the corresponding author upon reasonable request.
