# Supplementary material for: Probing Short‐Range Correlations in the van der Waals Magnet CrSBr by Small‐Angle Neutron Scattering
Source: Small Sci. 2024 Jun 13;4(8):2400244. doi: 10.1002/smsc.202400244 (PMC11935199; doi:10.1002/smsc.202400244)
Supplement: Supplementary file 1 — Supplementary Material [file SMSC-4-2400244-s001.pdf]

Supporting Information

Probing short-range correlations in the van der Waals magnet CrSBr by Small-Angle Neutron Scattering.

Andrey Rybakov, Carla Boix-Constant, Diego Alba Venero, Herre S. J. van der Zant, Samuel Mañas-Valero and Eugenio Coronado*

A. Rybakov, C. Boix-Constant, S. Mañas-Valero, E. Coronado
Instituto de Ciencia Molecular (ICMol), Universitat de València, Catedrático José Beltrán 2, Paterna, 46980 Spain.
E-mail: samuel.manas@uv.es

D. Alba Venero
ISIS Neutron and Muon Facility, Science and Technology Facilities Council, Rutherford Appleton Laboratory, Chilton OX11 0QX, United Kingdom.

H. S. J. van der Zant, S. Mañas-Valero
Kavli Institute of Nanoscience, Delft University of Technology, Lorentzweg 1, 2628 CJ Delft, The Netherlands.
E-mail: S.ManasValero@tudelft.nl

This file contains the **Supplementary Figures 1-16** and **Supplementary Table 1**.

Contents:

1. Experimental data. 2
   a. SANS signal. 2
   b. Integrated SANS signal (temperature dependence). 4
   c. Integrated SANS signal (field dependence). 5
2. Data analysis. 6
   a. I(T) analysis. 7
   b. I(T) – I(300 K) analysis. 11
3. Simulations. 15
4. Elastic SANS contribution. 19

1. Experimental data.

a. SANS signal.

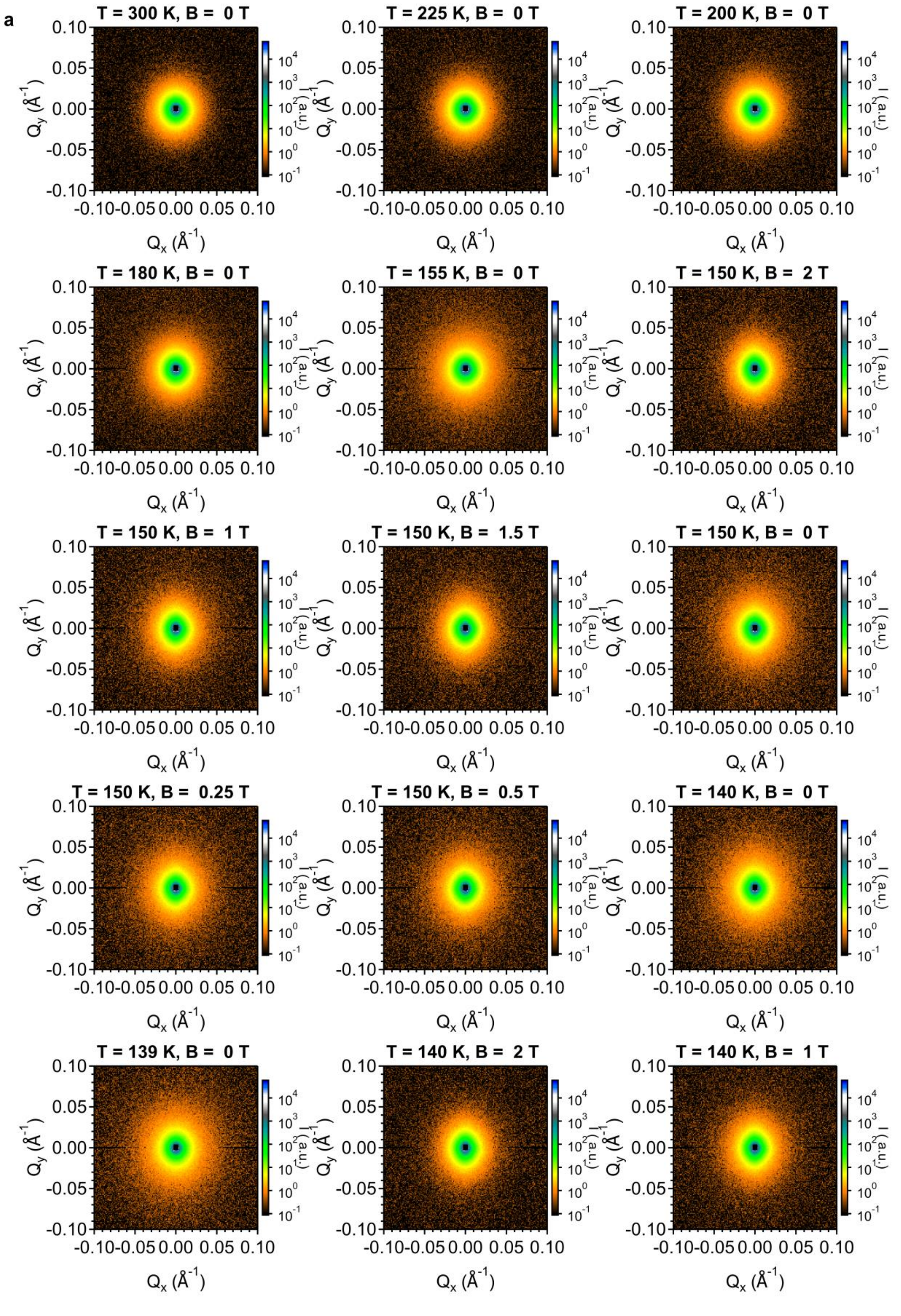

Supplementary Figure 1.- SANS signal at different temperatures and magnetic fields.

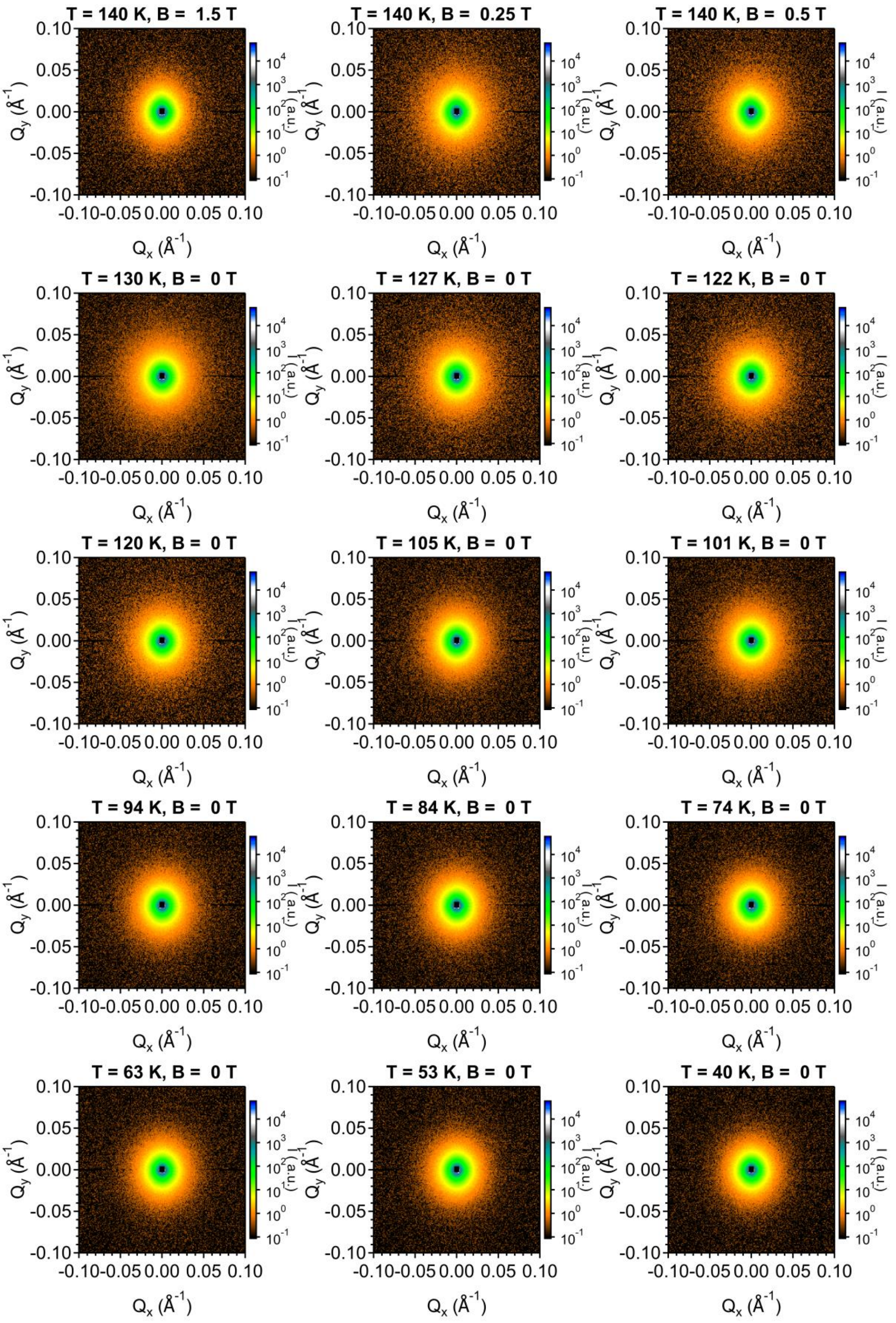

Supplementary Figure 1.- (continues from previous page) SANS signal at different temperatures and magnetic fields.

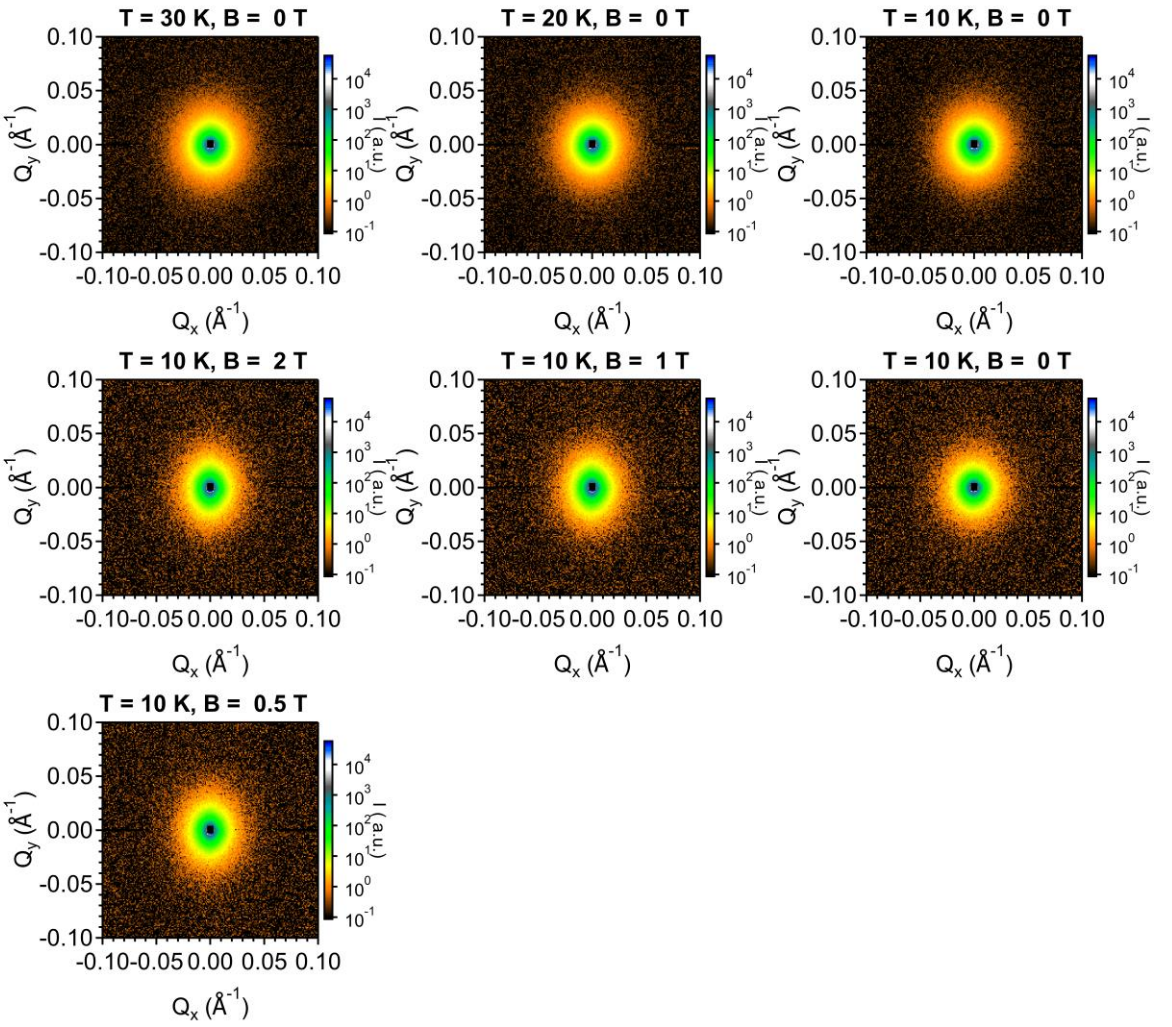

Supplementary Figure 1.- (continues from previous page) SANS signal at different temperatures and magnetic fields.

b. Integrated SANS signal (temperature dependence).

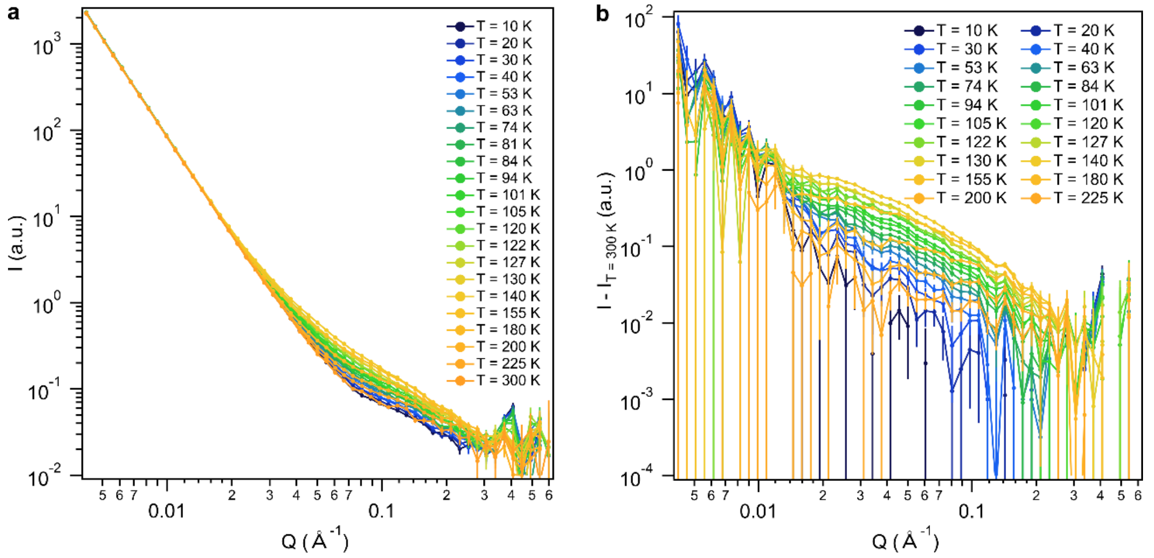

Supplementary Figure 2.- a) SANS signal at different temperatures. **b)** Magnetic contribution of the SANS signal after removing the structural component ($I_{T = 300\ K}$) at different temperatures.

c. Integrated SANS signal (field dependence).

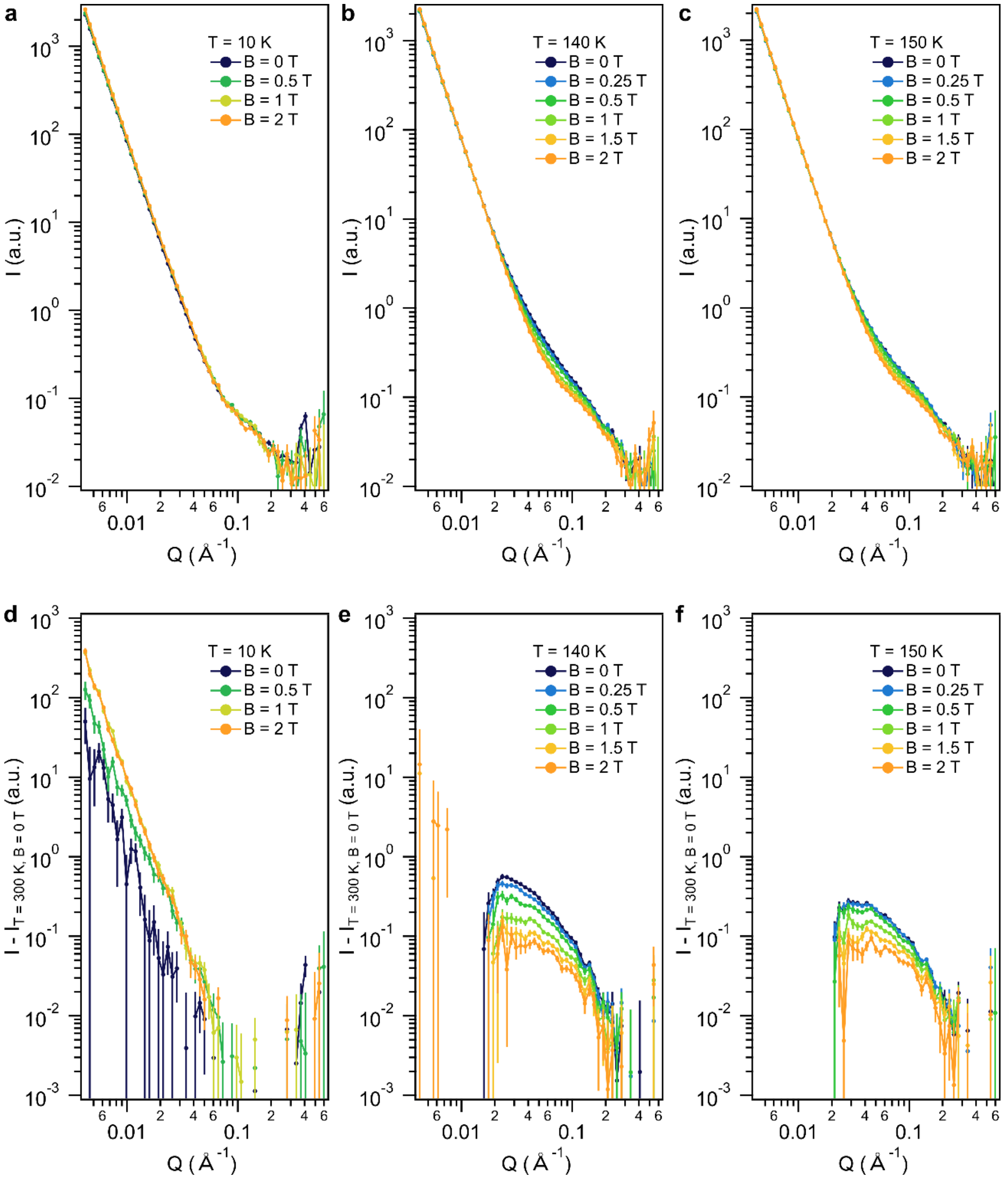

Supplementary Figure 3.- SANS signal (**a-c**) and magnetic contribution of the SANS signal (**d-f**) at different temperatures and magnetic fields.

2. Data analysis.

The data is fitted considering two different approaches. In the first approach (**Supplementary Section 2.a**), we fit the spectra at 300 K (high-temperature paramagnetic phase) to a power law, being $I_{T=300K}(Q) = \frac{I_{P,300K}}{Q^{4-n,300K}} + B_{300K}$, where I_P is a Porod scale term, n is an exponent and B is a Q-independent background constant. Then, we employ the relationship $I_{T\neq300K}(Q) = \frac{I_{OZ}(0)}{1+(\xi Q)^2} + \frac{I_{P,300K}}{Q^{4-n,300K}} + B$, where $I_{OZ}(0)$ is the Ornstein-Zernike intensity scaling and ξ is the correlation length. In the second approach (**Supplementary Section 2.b**), we consider $I(Q) - I_{300K}(Q) = \frac{I_{OZ}(0)}{1+(\xi Q)^2} + \frac{I_P}{Q^4} + B$. We note that both approaches are compatible between them, although the second one yields to a correlation length with larger error bars at low temperatures, that arise from an overparametrized fitting due to the absence of correlations within our experimental window range resolution. Despite being ξ constant below T_N, the absence of correlations is accounted by the suppression of $I_{OZ}(0)$. Fits shown in the main text are obtained with the first approach.

a. **I(T) analysis.**

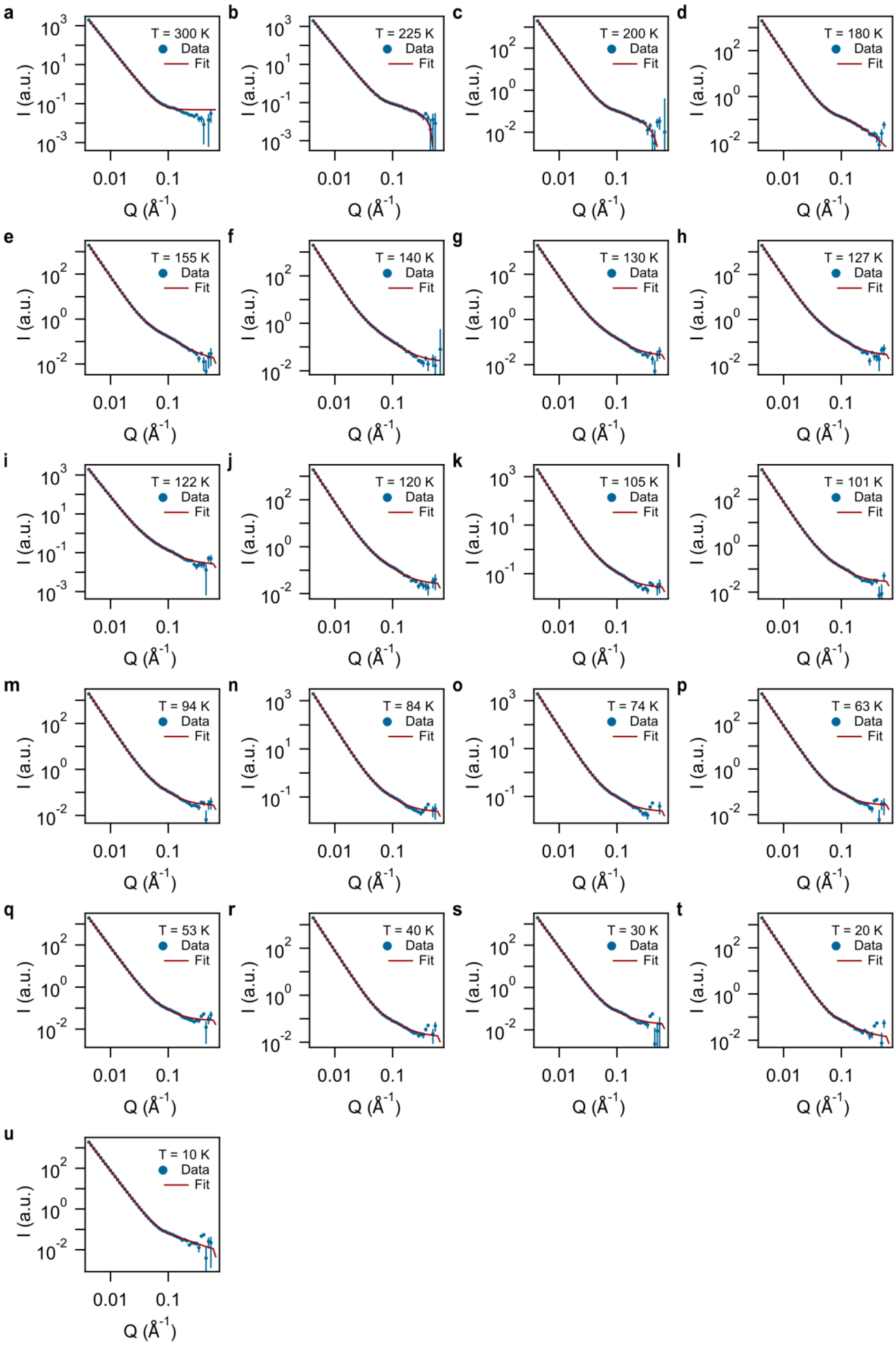

Supplementary Figure 4.- Fitted SANS signal at different temperatures (see Methods).

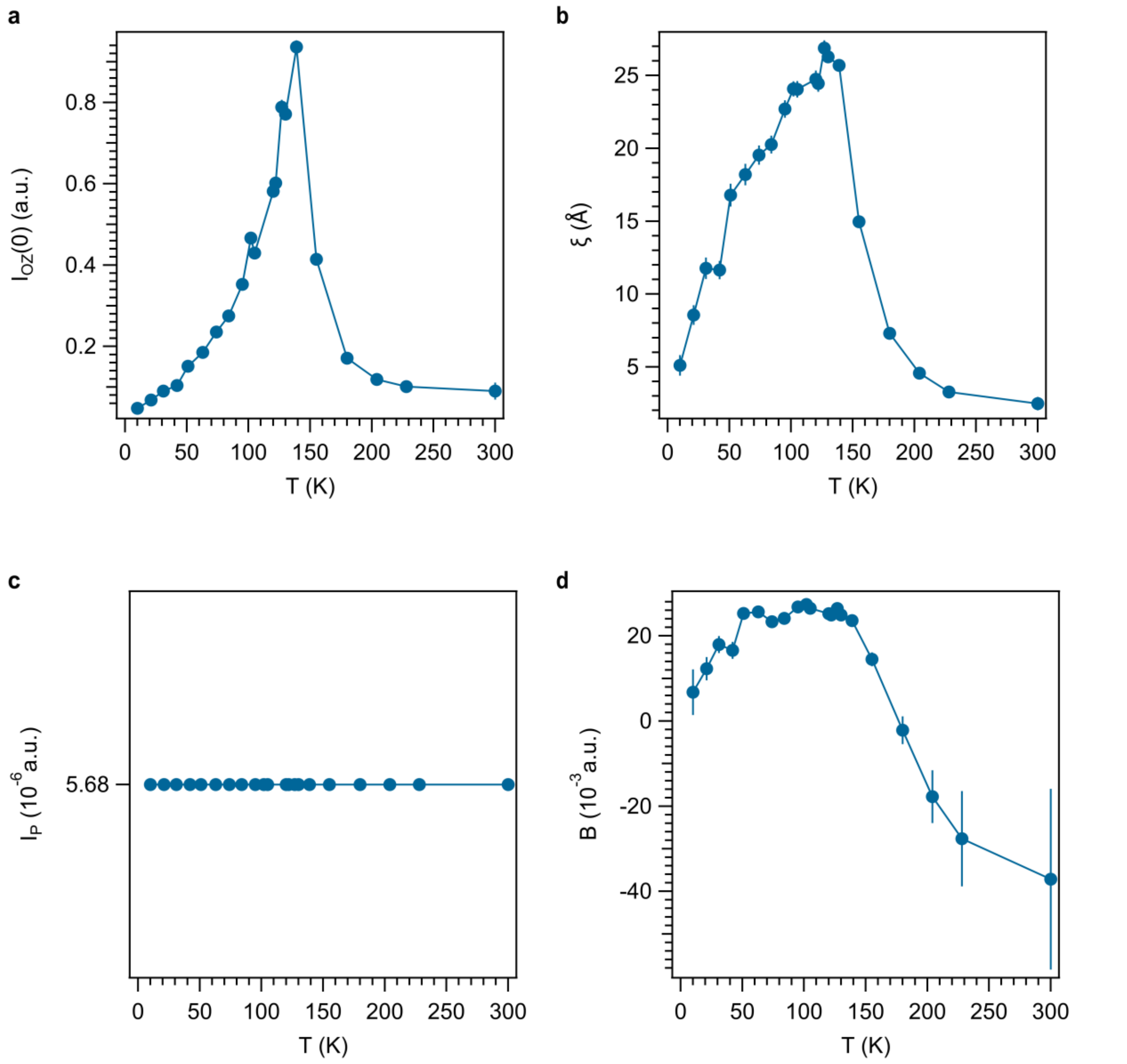

Supplementary Figure 5.- Thermal evolution of the intensity scale **(a)**, correlation length **(b)**, Porod scale factor **(c)** and background term **(d)** obtained from the fits shown in the **Supplementary Figure 4**.

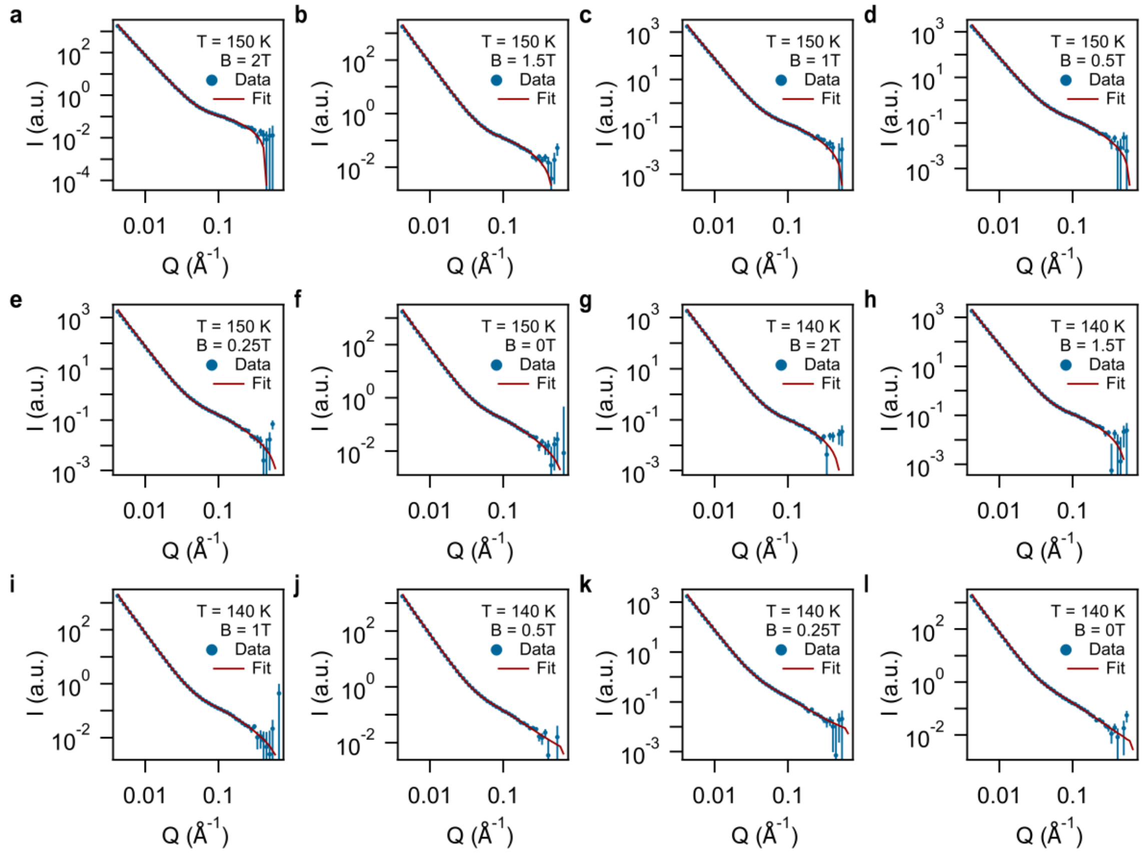

Supplementary Figure 6.- Fitted SANS signal at different temperatures and fields (see Methods).

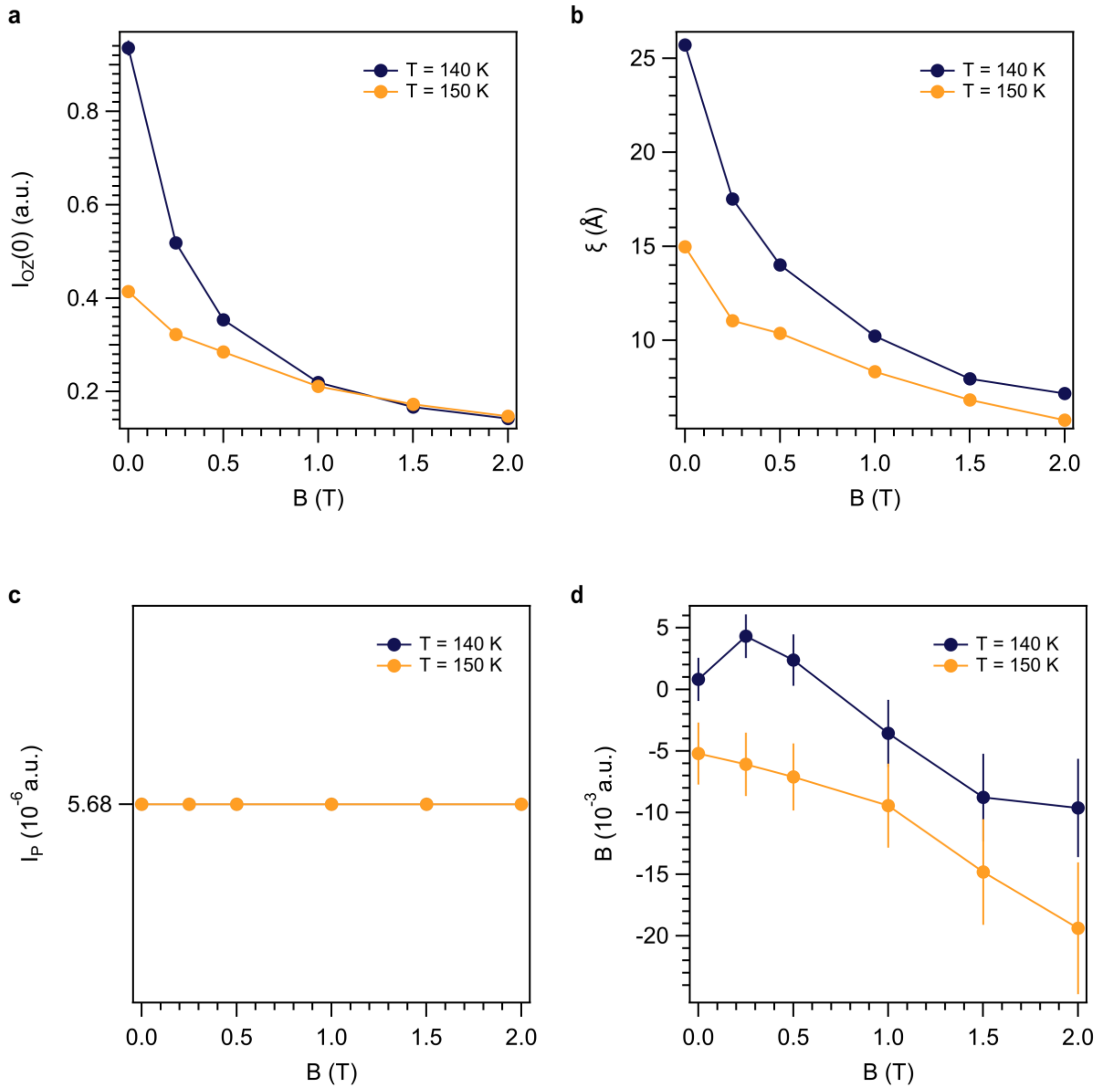

Supplementary Figure 7.- Thermal evolution of the intensity scale **(a)**, correlation length **(b)**, Porod scale factor **(c)** and background term **(d)** obtained from the fits shown in the **Supplementary Figure 6**.

b. I(T) – I(300 K) analysis.

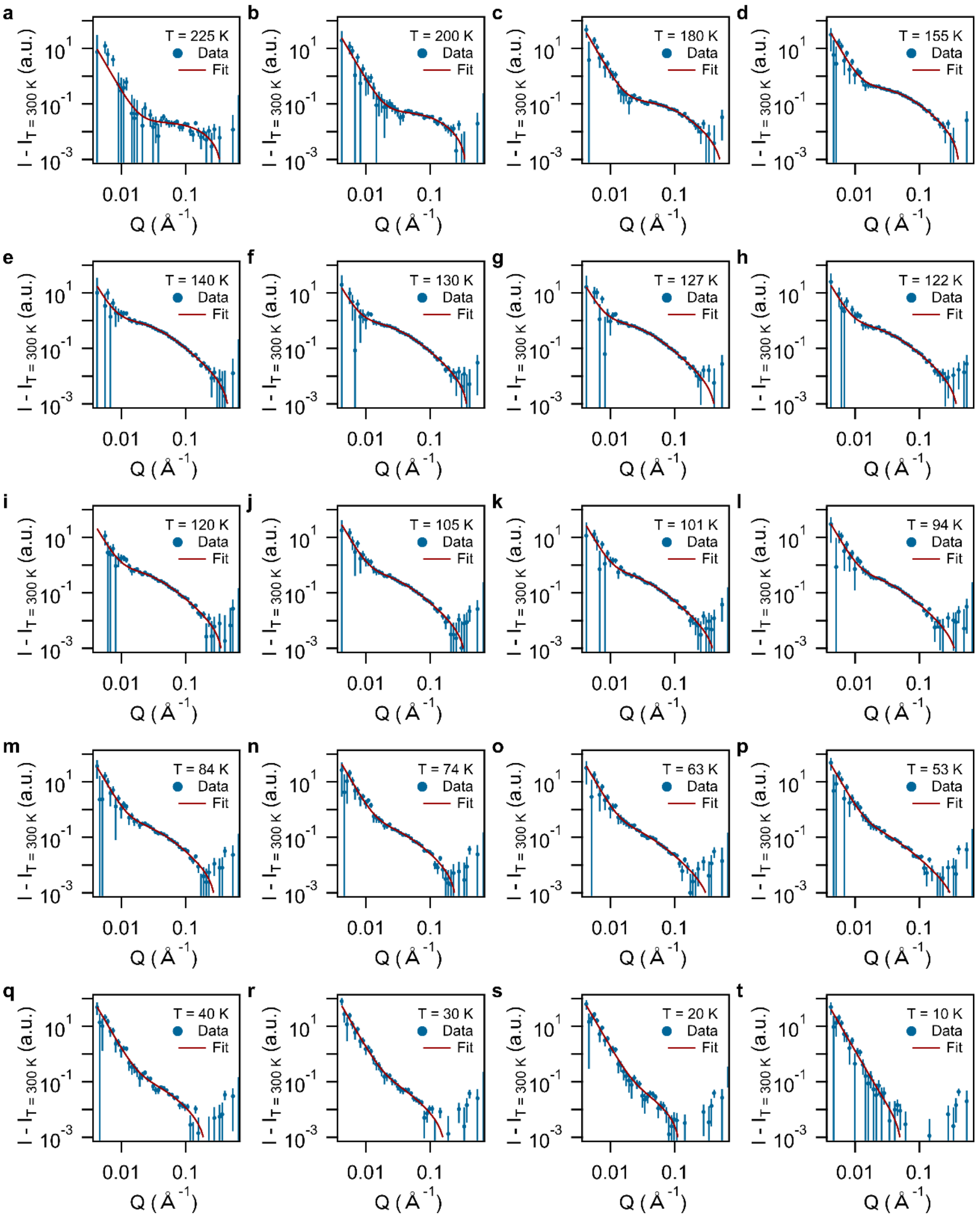

Supplementary Figure 8.- Magnetic contribution of the SANS signal fitted following an Ornstein-Zernike law plus a Porod term and background at different temperatures.

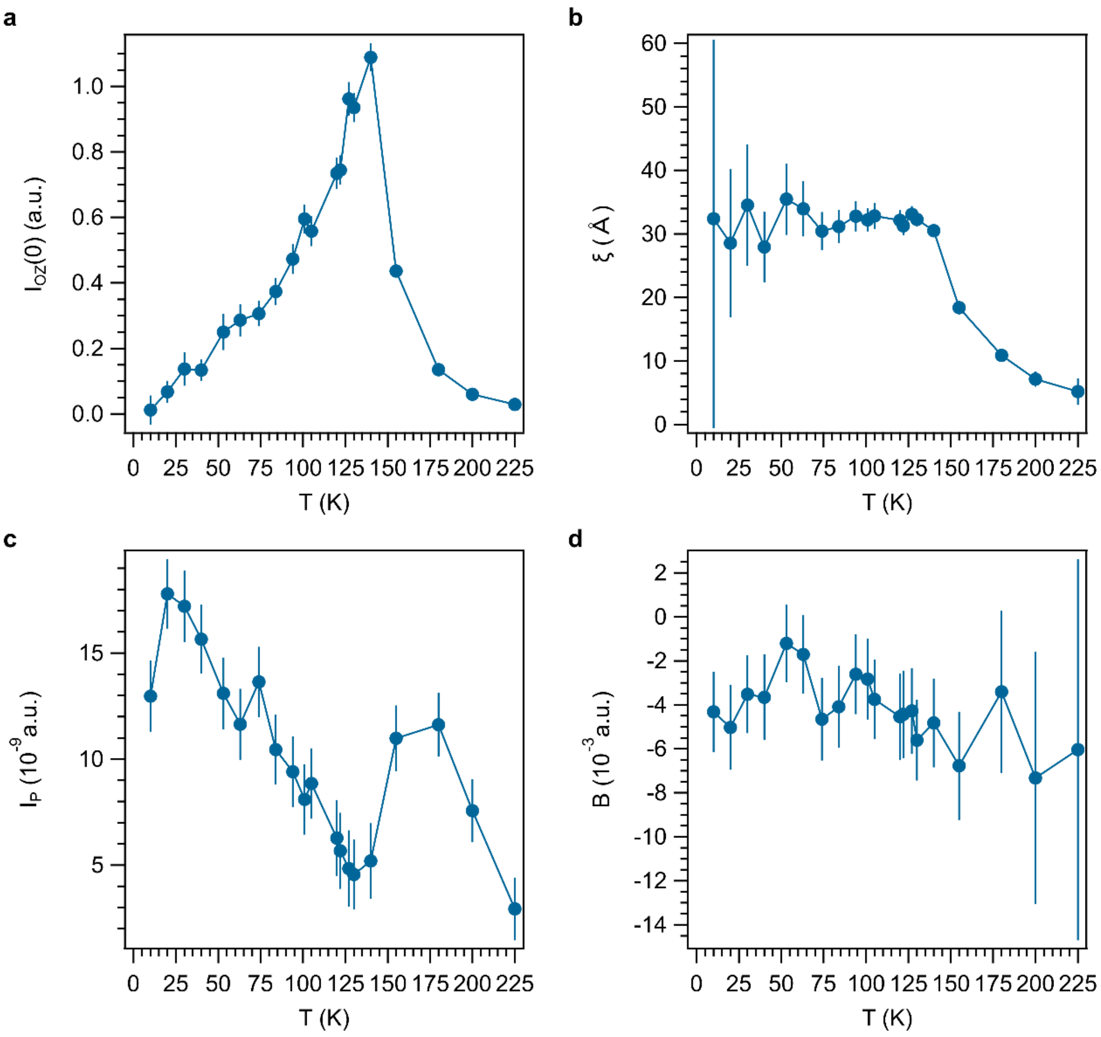

Supplementary Figure 9.- Thermal evolution of the intensity scale **(a)**, correlation length **(b)**, Porod scale factor **(c)** and background term **(d)** obtained from the fits shown in the **Supplementary Figure 8**.

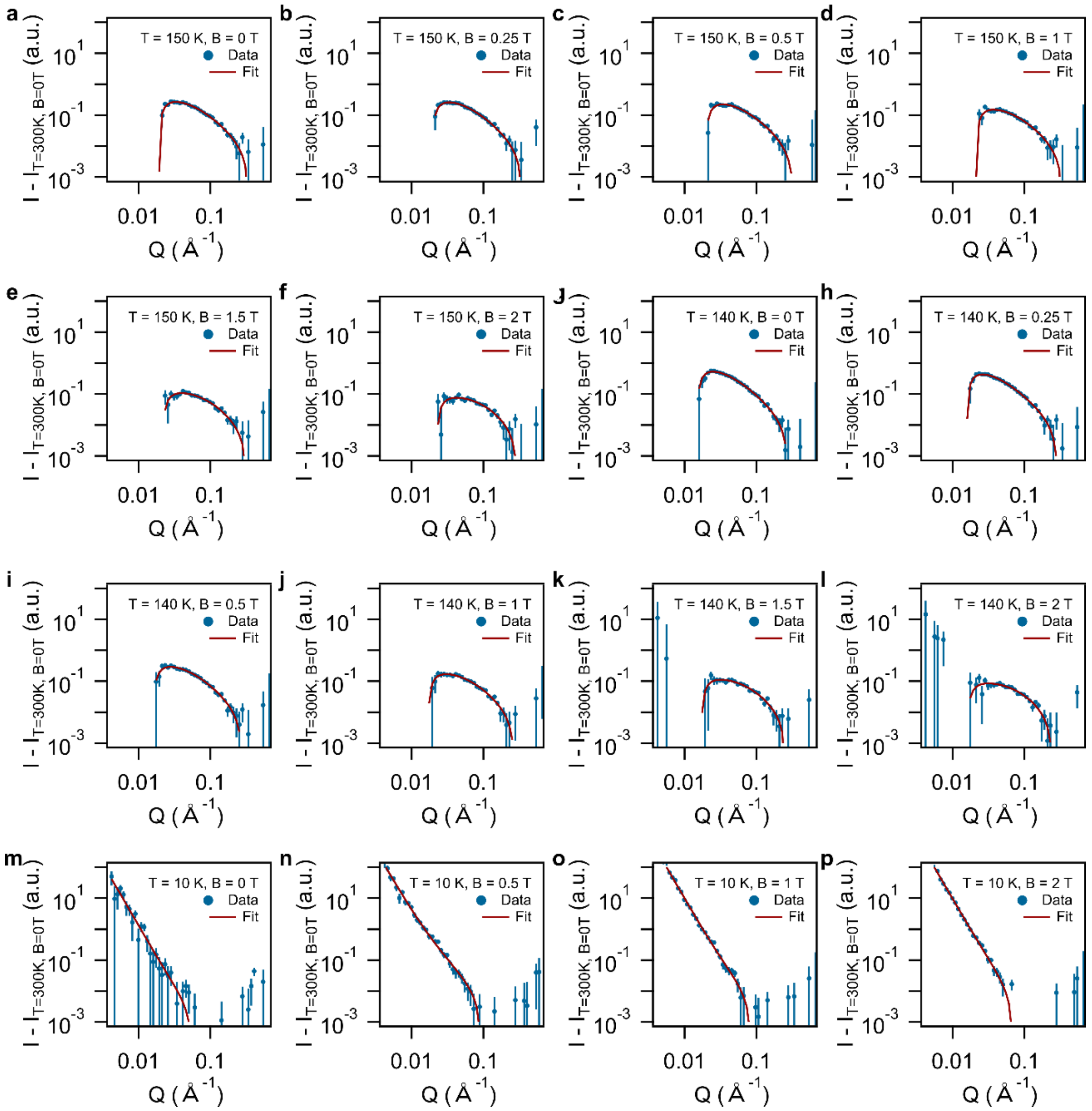

Supplementary Figure 10.- Magnetic contribution of the SANS signal fitted following an Ornstein-Zernike law plus a Porod term and background at different temperatures and magnetic fields.

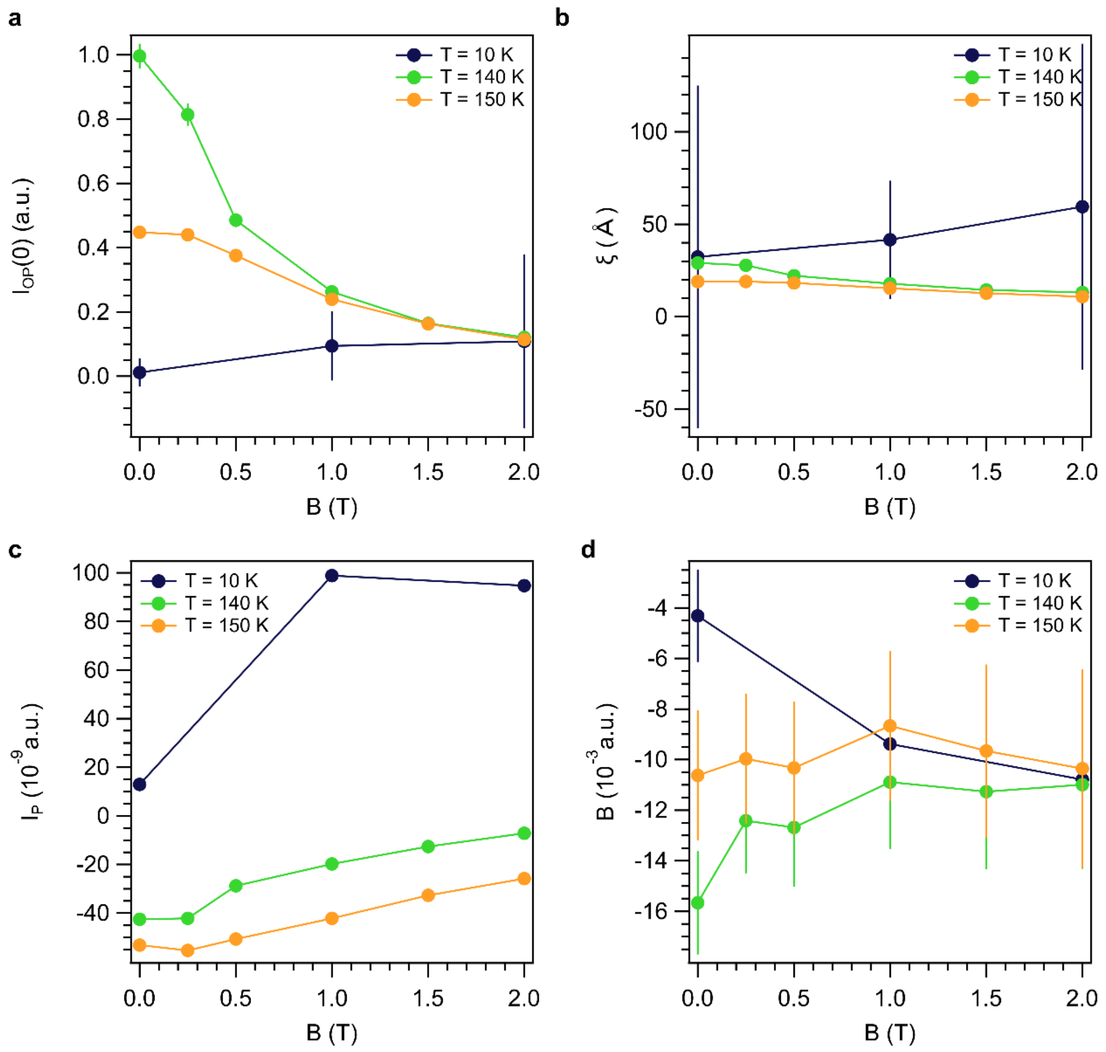

Supplementary Figure 11.- Field evolution of the intensity scale **(a)**, correlation length **(b)**, Porod scale factor **(c)** and background term **(d)** at different temperatures obtained from the fits shown in the **Supplementary Figure 10**.

3. Simulations.

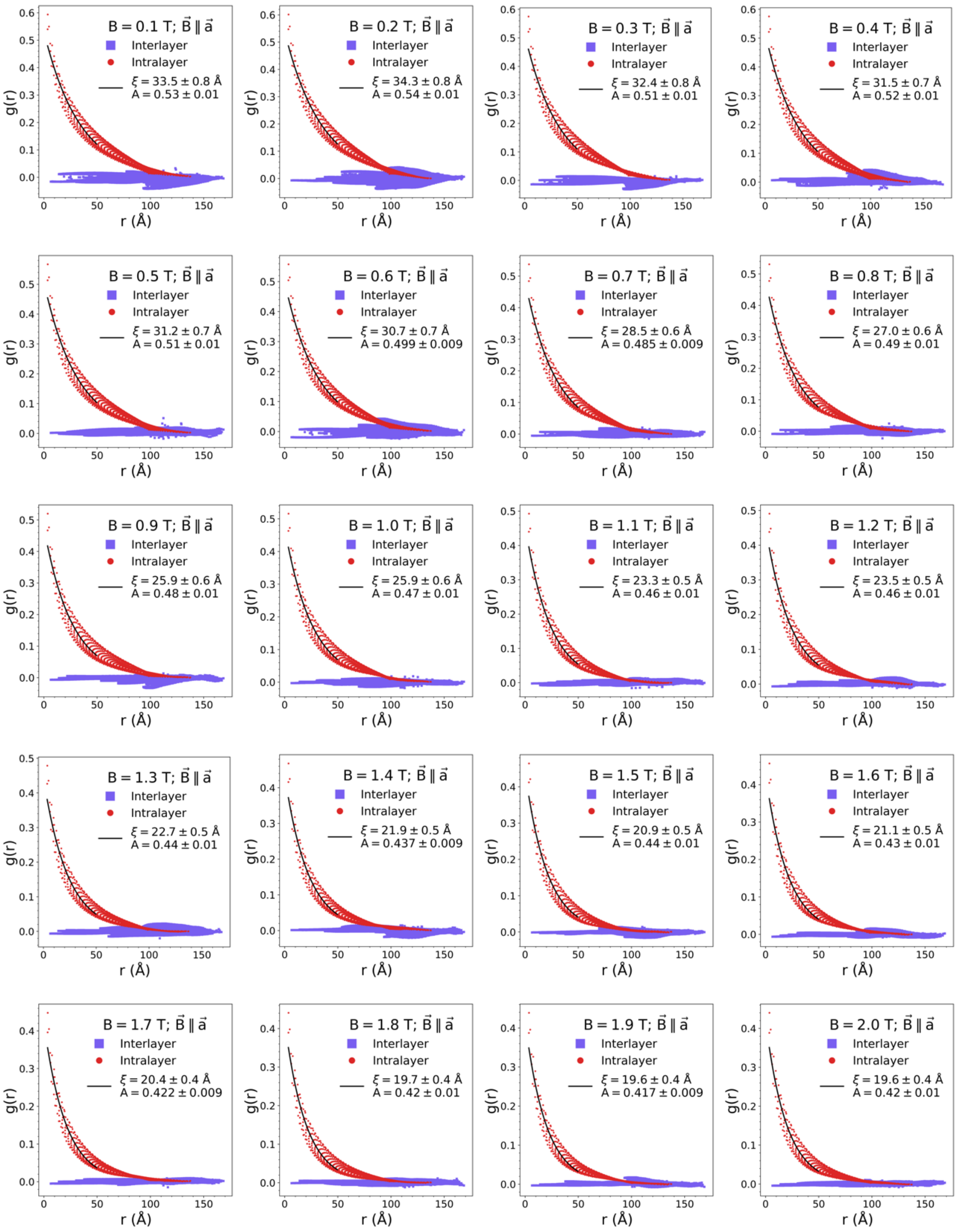

Supplementary Figure 12.- Distance-averaged dynamic spin-spin correlation function and exponential fit to $A \cdot e^{-r/\xi}$ for the intralayer part of the correlation function for different values of magnetic field at T = 140 K. Magnetic field is oriented along the direction of crystallographic axis *a*. The spin pairs are separated into two groups: interlayer (spin pairs where both spins belong to different layers) and intralayer (spin pairs where both spins belong to the same layer).

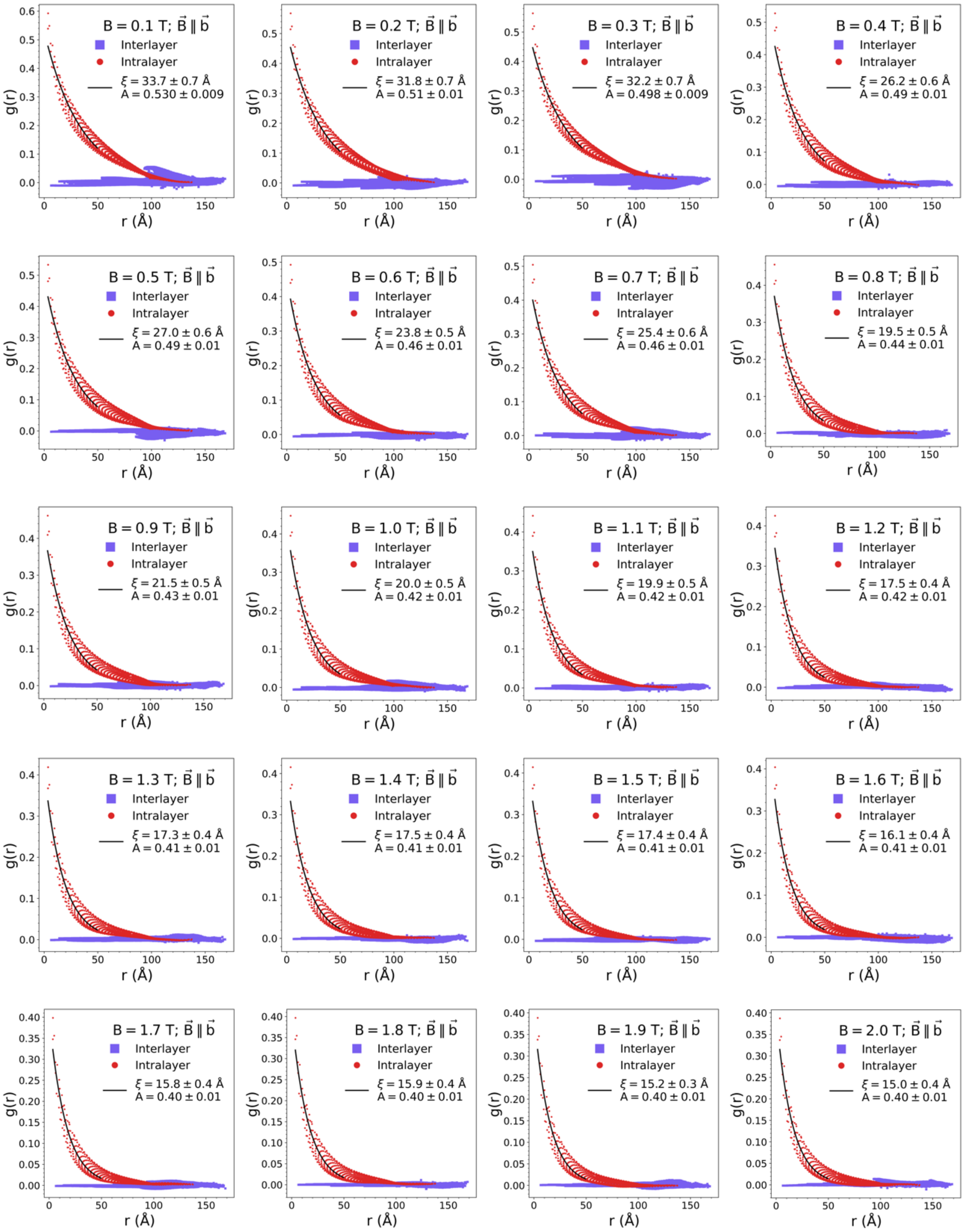

Supplementary Figure 13.- Distance-averaged dynamic spin-spin correlation function and exponential fit to $A \cdot e^{-r/\xi}$ for the intralayer part of the correlation function for different values of magnetic field at T = 140 K. Magnetic field is oriented along the direction of crystallographic axis *b*. The spin pairs are separated into two groups: interlayer (spin pairs where both spins belong to different layers) and intralayer (spin pairs where both spins belong to the same layer).

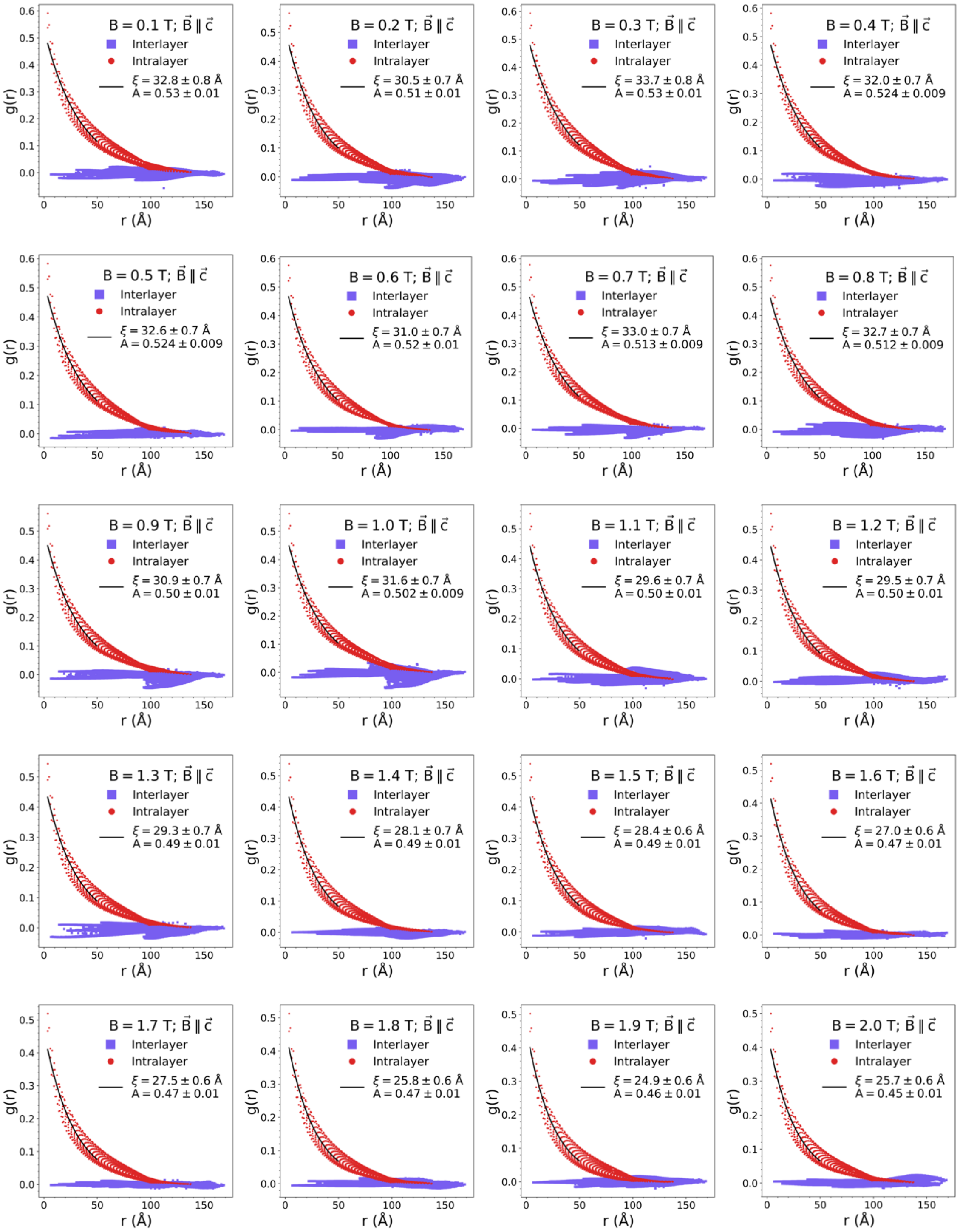

Supplementary Figure 14.- Distance-averaged dynamic spin-spin correlation function and exponential fit to $A \cdot e^{-r/\xi}$ for the intralayer part of the correlation function for different values of magnetic field at T = 140 K. Magnetic field is oriented along the direction of crystallographic axis *c*. The spin pairs are separated into two groups: interlayer (spin pairs where both spins belong to different layers) and intralayer (spin pairs where both spins belong to the same layer).

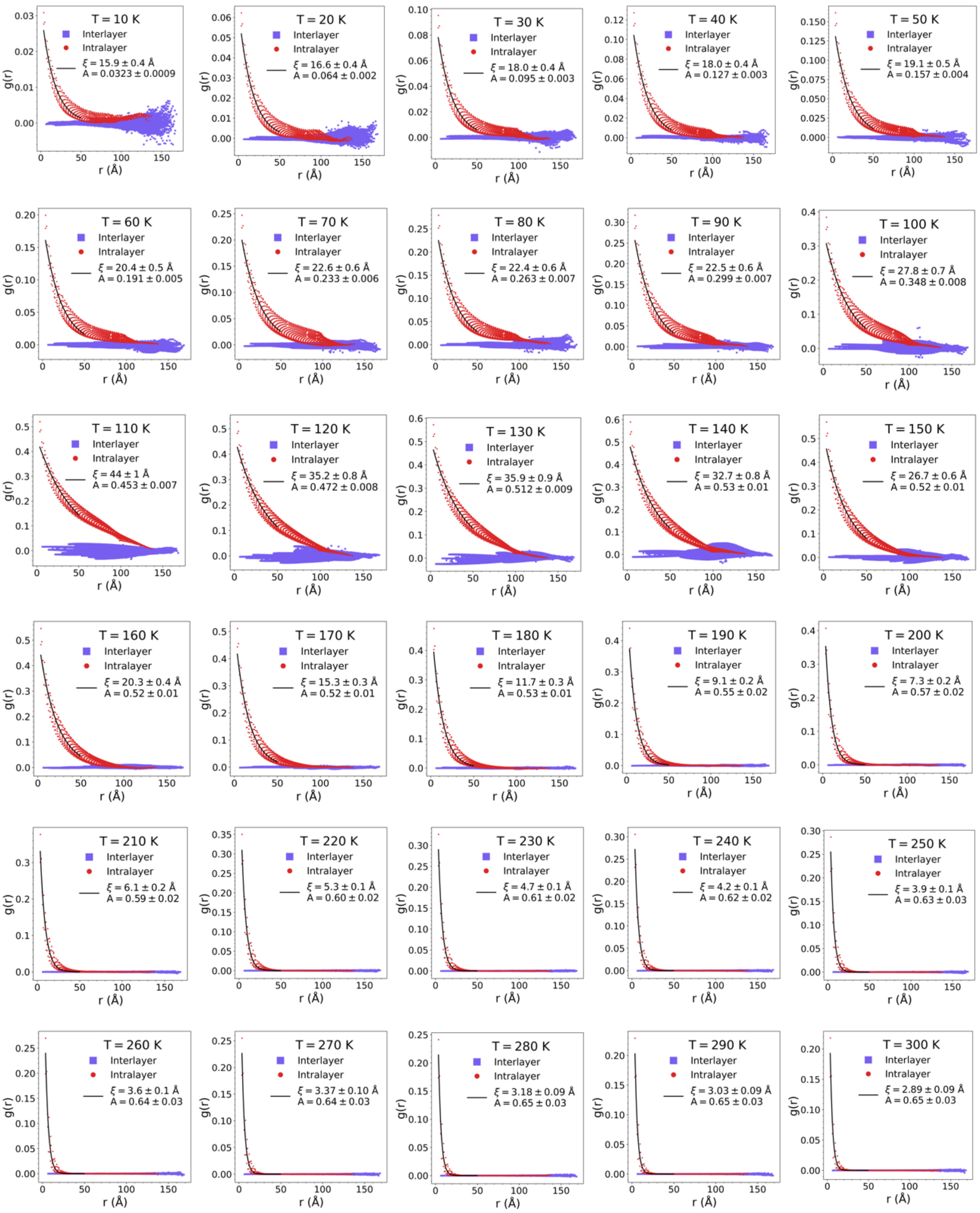

Supplementary Figure 15.- Distance-averaged dynamic spin-spin correlation function and exponential fit to $A \cdot e^{-r/\xi}$ for the intralayer part of the correlation function at different temperatures and zero applied magnetic field. The spin pairs are separated into two groups: interlayer (spin pairs where both spins belong to different layers) and intralayer (spin pairs where both spins belong to the same layer).

Supplementary Table 1.- Parametrization of the spin Hamiltonian. The notation is the one of the Hamiltonian from the main text: spins are not normalized; double counting is included. We provide unscaled parameters in this table. Actual parameters used in simulations are those multiplied by the factor 1.5.

Intralayer

Neighbor's order	1	2	3	4	5	6	7
$J_{isotropic}$, meV	-0.95	-1.69	-0.835	-0.045	-0.045	0.185	-0.145
DMI, meV	(0, 0.07, 0)	-	(0.18, 0, 0)	-	-	-	-

Interlayer

Neighbor's order	1	2
$J_{isotropic}$, meV	0.0008	-0.0008

SIA anisotropy

A_{xx}, meV	-0.018
A_{yy}, meV	-0.034
A_{zz}, meV	0.018

4. Elastic SANS contribution.

We note that there is certainly an inelastic contribution to our signal, as expected as well in a reactor-based source (where the intensity is integrated without analyzing the energy). Nonetheless, as the neutron's speed is smaller than the speed of sound in the solid, the phonon contribution can be considered negligible (see eq. 3.43 from A. Michels and J. Weissmüller, Rep. Prog. Phys. 71 066501, 2008). It is more challenging to assess the magnon contribution. However, we do not observe any significant variation upon different wavelengths (as expected for an inelastic contribution), thus indicating that the signal is mostly elastic (A. Michels and J. Weissmüller, Rep. Prog. Phys. 71 066501, 2008), as shown in the **Supplementary Figure 16**.

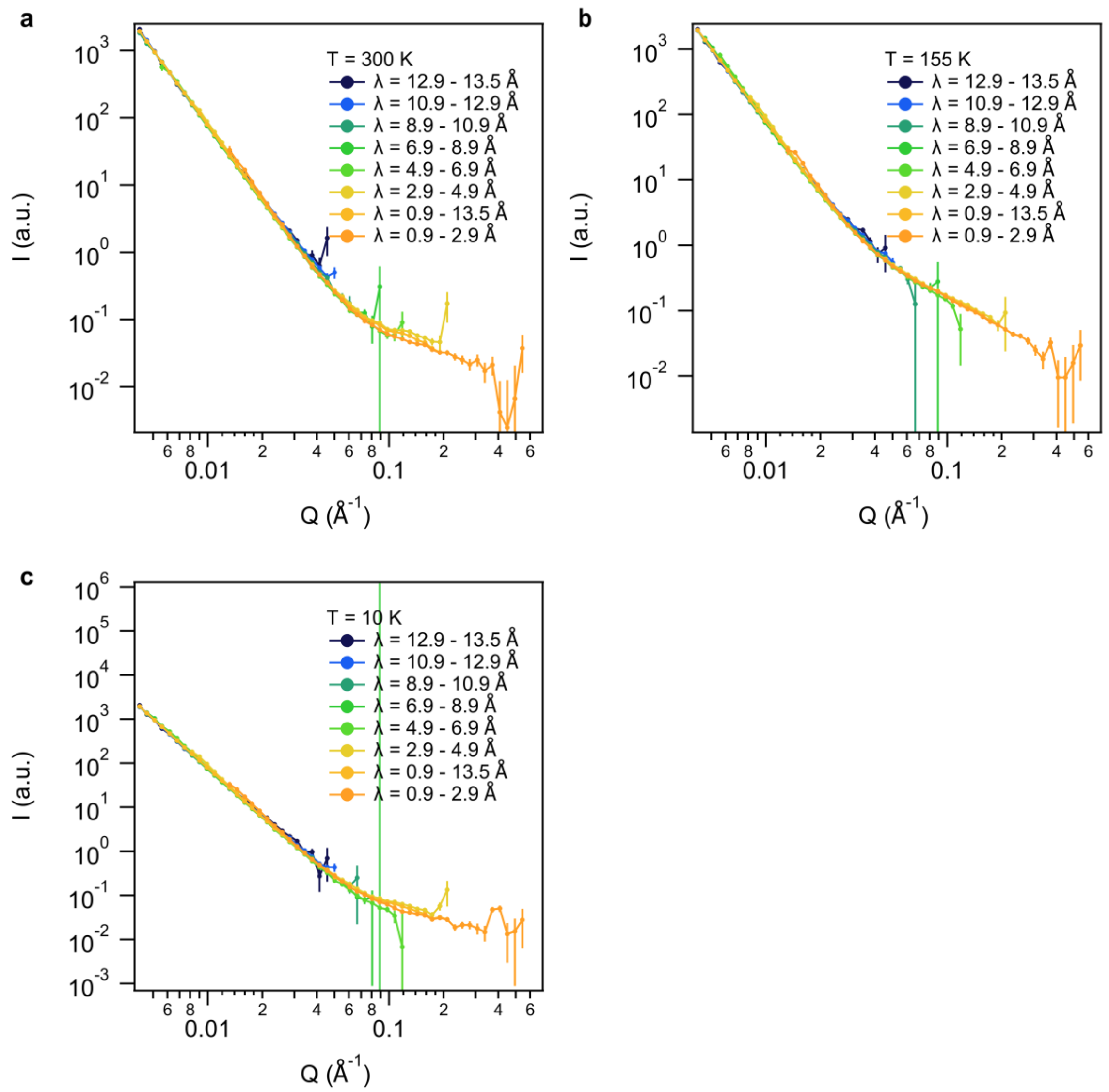

Supplementary Figure 16.- Selected 1D data reduction using different sections of the incoming wavelength spectrum. It can be seen that the overlap between the curves is very good, indicating the scattering intensity is only dependent on the momentum transfer of the neutrons.
